# Genes, Culture and Conservatism-A Psychometric-Genetic Approach

**DOI:** 10.1007/s10519-015-9768-9

**Published:** 2015-11-20

**Authors:** Inga Schwabe, Wilfried Jonker, Stéphanie M. van den Berg

**Affiliations:** Department of Research Methodology, Measurement, and Data Analysis, University of Twente, Drienerlolaan 5, 7522 NB Enschede, The Netherlands

**Keywords:** Conservatism, Liberalism, Genotype–environment interaction, Measurement error, Psychometrics, IRT

## Abstract

The Wilson−Patterson conservatism scale was psychometrically evaluated using homogeneity analysis and item response theory models. Results showed that this scale actually measures two different aspects in people: on the one hand people vary in their agreement with either conservative or liberal catch-phrases and on the other hand people vary in their use of the “?” response category of the scale. A 9-item subscale was constructed, consisting of items that seemed to measure liberalism, and this subscale was subsequently used in a biometric analysis including genotype–environment interaction, correcting for non-homogeneous measurement error. Biometric results showed significant genetic and shared environmental influences, and significant genotype–environment interaction effects, suggesting that individuals with a genetic predisposition for conservatism show more non-shared variance but less shared variance than individuals with a genetic predisposition for liberalism.

**Electronic supplementary material:**

The online version of this article (doi:10.1007/s10519-015-9768-9) contains supplementary material, which is available to authorized users.

## Introduction

The term conservatism is used in many ways (Pedhazur and Schmelkin 1991), but most often refers to politic-economic conservatism. More generally, conservatism can be seen as a generalized resistance to change and ambiguity which is expressed as a preference for safe, traditional and conventional forms of institutions and behaviour. Wilson and Patterson (1968) developed a conservatism scale to measure social attitudes related to the conservative personality. They regarded the then existing scales to be of poor psychometric quality because of susceptibility to agreement-response bias and complex, double-barrelled and/or confusing questions. Instead, their conservatism scale consists of very short catch-phrases. Examples of catch-phrases include “Liberals” and “Living together”. The test taker is then asked “Please indicate whether or not you agree with each topic by circling “Yes” or “No” as appropriate. If uncertain please circle “?”. These catch-phrases were expected to activate the respondent’s affective system, the system Wilson and Patterson (1968) hypothesised to be the most influential component for conservative attitudes and behaviours. The affective system seems indeed to be important, since conservatives tend to have stronger disgust reactions than liberals (Inbar et al. 2009) and brain data suggest that conservatives and liberals process risk and fear differently (Schreiber et al. 2013). Furthermore, Hibbing et al. (2014) found that conservatives tend to have stronger physiological responses to features of the environment that are negative and also devote more psychological resources to these stimuli.

The development of the scale was based on seven characteristics that Wilson and Patterson (1968) expected to be present in highly conservative individuals: (1) religious fundamentalism, (2) right-wing political orientation, (3) insistence on strict rules and punishments, (4) intolerance of minority groups, (5) preference for conventional art, clothing and institutions, (6) anti-hedonistic outlook, and (7) superstition and resistance to science. A large pool of more than 130 catch-phrases were created that Wilson and Patterson (1968) regarded to be effective discriminators for these seven characteristics. Based on three successive item analyses (Wilson and Patterson^1968^), 50 items were selected from this pool. To control for response bias, half of the items were phrased in the affirmative direction of conservatism and half of the items were liberally phrased. Although initially conceived of as a unidimensional scale (Wilson 1973), subsequent research on the structure of the scale showed that four factors were required to explain most of the observed variance. These factors were named (1) militarism-punitiveness (12 items), (2) anti-hedonism (12 items), (3) ethnocentrism and out-group hostility (12 items) and (4) religion-puritanism (12 items; Wilson 1973). Eaves et al. (1999) devised a shortened and somewhat altered conservatism scale consisting of 28 items. Most of the items were taken from the original conservatism scale, with a few items added that were regarded relevant at the time of data collection. The eigenvalues of the inter-item correlations for the 28 items suggested, according to Eaves et al. (1999), that a general “conservative - liberalism” factor was substantial but not exhaustive to explain the observed variance. An exploratory factor analysis with oblique rotation suggested that five factors explained most of the observed variance in 24 of the 28 items. These factors were named: (1) sexual permissiveness (8 items), (2) economic liberalism (5 items), (3) militarism (5 items), (4) political preference for democrats or republicans (2 items) and (5) religious fundamentalism (5 items). Note that in all these psychometric analyses, linear relationships were assumed between the items, treating the “?” response as exactly midway between a “yes” and “no” answer.

### Prior genetic research

Using various versions of the Wilson and Patterson conservatism scale, research has shown that both, genetic and cultural, influences are responsible for the observed variance in conservatism. Based on a conservatism measure derived from the original scale (Wilson and Patterson 1968), Martin et al. (1986) reported monozygotic (MZ) twin correlations of 0.60 for males and 0.64 for females, assessed in a large sample from the Australian Twin Registry. Eaves et al. (1997) reported on MZ and dizygotic (DZ) twin correlations across age (9.5−75 years). They found that prior to age 20 all variance due to individual differences is age-related, implicating environmental influences. However, after age 20, age effects vanished and there were significant differences between MZ and DZ twin correlations, suggesting genetic influences. In a later study, Eaves et al. 1999 reported heritability estimates of 0.65 for males and 0.45 for females based on the Virginia 30,000 study of twins and their relatives. Bouchard et al. (2003) assessed the 28-item conservatism scale in the Minnesota Study of Twins Reared Apart (MISTRA) and reported a heritability of 0.56. Hatemi et al. (2014) published results of a genome-wide association study (GWAS) meta-analysis, where several cohorts and, among other measures, various versions of the Wilson−Patterson conservatism scale were used. They also reported on variance components. They found a combined weighted mean of relative influences across measures and cohorts of 0.40 for genetic influences, 0.18 for common-environmental influences and 0.42 for unique-environmental influences. The GWAS meta-analysis showed no genome-wide significant hits, which may be partly related to the heterogeneity of the measures used across cohorts, but could also be due to multidimensionality of the conservatism measure (van der Sluis et al. 2010).

### Need for psychometric evaluation

Establishing a measure with good psychometric properties is important for finding genomic signals for personality traits such as conservatism (van der Sluis et al. 2010; van den Berg and Service 2012). Prior research on the psychometric dimensionality of the conservatism scale was based on linear factor analysis with “yes”, “?” and “no” coded as 3, 2 and 1 respectively, thus assuming that a “?” response is exactly midway between a “yes” and “no” response. This assumption, however, is not necessarily true—a “?” response might mean something else than being psychologically (exactly) between a “yes” and “no” answer. Reactions like “I don’t know what I think” or “I don’t know what is meant by busing”, are psychologically different from for example an “I don’t care” reaction, or a reluctance to convey the true affective response. Converse (1964) and other political scientists (e.g. Campbell et al. 1960) have demonstrated that the general American public is largely uninformed about current political affairs and has gaps in knowledge of political systems. Arguably, it is likely that respondents do not understand or care about (some of) the catch-phrases of the Wilson−Patterson scale.

In addition, it is important to know to what extent scores on this scale reflect true trait variability and to what extent they reflect measurement error. Measured as split-half internal consistency, the conservatism scale has been reported to have a high reliability of 0.94 (Wilson and Patterson 1968). This finding is supported by several studies. For example, Henningham (1996) reports alpha reliability of 0.81 on a 27-item version based on the original scale and an alpha reliability of 0.74 on a simplified and modernized 12-item version. In this paper, the psychometric properties of the conservatism scale were assessed more rigorously by using homogeneity analysis (de Leeuw and Mair 2009) and item response theory (IRT), thereby greatly relaxing the assumption of prior research of linear relationships among items. With the establishment of a good scale, a biometric analysis including genotype–environment interaction was done.

### Genotype–environment interaction

Genotype–environment interaction refers to the situation that some genotypes are more sensitive to changes in the environment than other or, conversely, that genotypes respond differently to the same environment (see e.g. Cameron 1993; Martin 2000; Sorensen 2010). Although various studies suggest that genotype–environment interaction is an important phenomenon in complex behavioural traits (e.g., anti–social behaviour, Caspi et al. 2002; cognitive ability, Turkheimer et al. 2003 or depression, Hicks et al. 2009), research on genotype–environment interaction has not been a focus of genetic studies on conservatism. Present study was concerned with an omnibus test to assess whether there is any statistically significant genotype–environment interaction. Therefore, the method that we use here to model genotype–environment interaction is parametrized such that both, genetic as well as environmental, influences are modelled as latent (i.e., unmeasured) variables. If indeed, genotype–environment interaction is found, future research on the etiology of conservatism can focus on the exact nature of this effect by collecting specific, environmental measures at the family or individual level, depending on the results of this research.

Schwabe and van den Berg (2014; see also Molenaar and Dolan 2014) recently developed a method that models genotype–environment interaction in such a way that statistical findings are independent of scale properties. This means that as long as a set of items measures a particular trait, such as conservatism, biometric results (i.e., conclusions regarding heritability and genotype–environment interactions) are the same regardless what particular (sub)set of items is used. This is important since it is generally recognized that statistical findings regarding non-linear effects such as genotype–environment interaction are dependent on the scale at which the analysis takes place; a simple transformation such as taking the logarithm or computing the root of a particular measure (e.g., a sum score) either obscures or reveals interaction effects (see e.g. Eaves et al. 1977; Martin 2000; van der Sluis et al. 2006; Eaves 2006; Molenaar et al. 2012; Schwabe and van den Berg 2014; Molenaar and Dolan 2014). Schwabe and van den Berg (2014; see also Molenaar and Dolan 2014) showed that the skewness in the phenotype distribution in large part determines finding a genotype–environment effect, even when that skewness in sum scores is only due to response frequencies in the items’ response categories. For instance, a relatively large proportion of “yes” responses on dichotomous yes–no questions leads to a skewed distribution of the *number* of “yes” answers (total test score). Slightly rephrasing the questions might cause no real change in item content (e.g., changing “Do you like peanut butter?” into “Do you like peanut butter very much?”), but can cause a change in proportion of yes-answers and thus change the skewness of the test score. Therefore, a rewording can lead to obscuring or revealing genotype–environment interaction effects even when the measured construct is the same. By applying the method by Schwabe and van den Berg 2014, that involves item-response theory (IRT) modelling while modelling genotype–environment interaction at the level of the latent construct, our results regarding genotype–environment are free of any statistical artefacts due to response category frequencies. Still, the scale at which we model the interaction effect is arbitrary, but at least it is identified by using an IRT model. This makes our results comparable to other studies with perhaps slightly different items or a subset of the items, but where the scale was identified in the same way (i.e. the same IRT model).

### This research

The first part of this study consists of a psychometric evaluation of the 28-item conservatism scale as used in Eaves et al. (1997, 1999), Bouchard et al. (2003) and the adult cohort in Hatemi et al. (2009). Item response models were used that take into account the categorical nature of the responses by modelling non-linear relationships between item responses and the trait being measured. In an exploratory analysis, multidimensional homogeneity models (Gifi 1990) that assume nominal response categories were fitted in order to re-evaluate the psychometric dimensionality of the Wilson−Patterson scale. The Gifi method relaxes the assumption that an “?” answer falls exactly halfway between a “yes” and a “no” answer. Based on the results, a new scale was devised. IRT models were then used to confirm the results of the homogeneity analysis and to evaluate the psychometric quality of the new scale. In the second part of this study, the new scale was used to investigate genotype–environment interaction. For the genotype–environment analysis, a Bayesian approach was used in which the biometric model and an IRT model were fitted simultaneously.

## Method

### Data

The data come from the Health and Life-Style Survey for Twins assessed in the Virginia 30K sample (Eaves et al. 1999; Hatemi et al. 2009), selecting data on twins and their parents. Part of this survey was the 28-item scale described above. Zygosity status was based on self-reported resemblance with a reported percentage correct of 95 % (Eaves et al. 1999). Total sample size was 14454. Mean age was 52.13 ($SD = 17.8$, range 16–94). For the psychometric analyses in the first part of this study, we used all available data from twins and their parents that had complete data for the 28 items ($N = 12315$, of which 10405 were twins). For the biometric modelling in the second part of this research we only used twin data (2795 MZ twin pairs, 3280 DZ twin pairs) and item data that was missing was assumed missing at random.

### Part I: psychometric analyses

For the psychometric analyses, only data from twins and their parents were used with complete data on all 28 items with “no” coded as 1, “?” coded as 2 and “yes” coded as 3. Items associated with conservatism [as reported by Eaves et al. (1999), i.e. items 1 (Death penalty), 9 (Military Drill, 10 (Draft), 16 (Capitalism), 17 (Segregation), 18 (Moral Majority), 20 (Censorship), 21 (Nuclear Power), 23 (Republicans), 25 (School Prayer) and 28 (Busing)] were reverse coded, so that a high sum score is associated with low conservatism (high liberalism). The analyses were done using SPSS (IBM 2013) and R (development core team 2007). R is an open source language and environment for statistical computing, which is freely available at http://cran.r-project.org

First, using SPSS (IBM 2013), a classical assessment of psychometric quality was performed on the scale as proposed by Eaves et al. (1999): computing item-total correlations and estimating reliability. Next, the responses were assumed nominal and a homogeneity analysis was done. Homogeneity analysis can be seen as a principal components analysis for nominal data. The analysis positions both individuals and item answer categories into one geometric space. It uses alternating least squares to minimize the distances between the position of an item’s particular answer category (“no”, “?”,“yes”) and individuals that chose that particular category (see e.g., Heiser and Meulman 1994; van der Kloot 1997). Using SPSS (IBM 2013), the dimensionality of the geometric space was determined. A two-dimensional homogeneity model was then further analysed with the R package homals (de Leeuw and Mair 2009). Based on the homogeneity analysis results, a unidimensional conservatism scale was constructed.

The reliability of the new scale was calculated in SPSS and IRT models were used to confirm the results of the homogeneity analysis and further evaluate the new scale. For the IRT modelling, the R package mirt (Chalmers 2012) was used. The IRT analysis was done using a generalized partial credit IRT model (GPCM) (Muraki 1992), which is an IRT model that is suitable for polytomous, ordinal data. The GPCM model has parameters both for difficulty (i.e., thresholds) as well as discrimination parameters that are the IRT analog of factor loadings. For our current data with three ordered response categories (“no”, “?”, “yes”) the GPCM specifies two threshold parameters for each item, one for the location on the scale where the probability of a “?” equals the probability of a negative response, and one parameter for the location on the scale where the probability of a positive response equals the probability of a “?” response. Also a Partial Credit Model (PCM) was applied, which is a restricted version of the GPCM where the discrimination parameters (factor loadings) are all assumed equal to 1. For model comparison purposes, the AIC and the BIC were computed. At item level, goodness of fit was evaluated using chi-square statistics, comparing observed and expected response frequencies for different bins of test scores.

### Part II: biometric analysis

In the second part of this study, the newly constructed scale was used in a biometric analysis including genotype–environment interaction. Here we follow the new method that integrates an IRT model into biometric modelling of genotype–environment interaction at the latent construct (see Schwabe and van den Berg 2014; Molenaar and Dolan 2014). As biometric model, the so-called ACE model was used which decomposes total phenotypic variance, $\sigma ^2_P$, into variance due to additive genetic influences ($\sigma ^2_A$), variance explained by common-environmental influences ($\sigma ^2_C$) and variance due to unique-environmental influences ($\sigma ^2_E$). Whereas common-environmental influences were parametrized to be perfectly correlated in a twin pair, we parametrized unique-environmental influences to be uncorrelated in one family.

*Bayesian approach*

van den Berg et al. (2007) showed that, in order to take full advantage of the IRT approach, both the IRT measurement model and the biometric model have to be estimated simultaneously, using a so called one-step approach. However, as this procedure is computationally burdensome, widespread methods of estimating variance components through structural equation modelling (SEM) reach their computational limit. van den Berg et al. (2007) showed that Bayesian statistical modelling can be an alternative to enable the simultaneous modelling of an IRT measurement and variance decomposition model. In the Bayesian approach, statistical inference is based on the posterior density of the model parameters which is proportional to the product of a prior probability and the likelihood function of the data (for further reading see e.g. Box and Tiao 1992). Here we use Gibbs sampling (Geman and Geman 1984; Gelfand and Smith 1990; Gelman et al. 2004), a Markov chain Monte Carlo (MCMC) algorithm, to study the posterior densities of model parameters. This method was applied using the freely obtainable MCMC software package JAGS (Plummer 2003). The JAGS script can be found in the online supplementary material. As similar syntax is used, the script can be used also in the free software package WinBUGS Lunn et al. (2000) with minor adaptations. As an interface from R to JAGS, the R package rjags was used (Plummer 2013).

As in Eaves and Erkanli (2003) and van den Berg et al. (2006, 2007), a Bayesian version of the ACE model was used that only specifies univariate distributions. This model is an extension of the Schwabe and van den Berg (2014) model to a (Generalized) Partial Credit model ((G)PCM, Muraki 1992) version at the measurement level. Furthermore, the model was extended to include, besides an interaction with unique-environmental influences, also an interaction with common-environmental influences, following Molenaar and Dolan (2014).

*Biometric and IRT model*

In the following, the full model, consisting of both variance decomposition (ACE model) and measurement model (IRT model), will be described for MZ and DZ twins.

We assumed that the additive genetic effect *A* and the common-environmental effect *C* are both normally distributed. Thus, we have for individual twin *j* from MZ twin pair *i*:

1 $$A_i\sim  N(0, \sigma ^2_A)$$

2 $$C_i\sim  N(0,\sigma ^2_{Ci})$$

3 $$\theta _{ij}\sim  N(A_i + C_i, \sigma ^2_{Ei})$$

where $\theta _{ij}$ is a person-specific latent variable that can be interpreted as the conservatism trait that is being assessed by the *k* items (i.e., the phenotype). To model genotype–environment interaction, we let the amount of variance due to environmental variance vary systematically with genotype *A*. Using this parametrization, we can distinguish between two different types of interaction effects. There can be an interaction with unique-environmental influences (henceforth referred to as A × E), but there can also be an interaction with common-environmental influences (henceforth referred to as A × C). To introduce A × C and A × E, $\sigma ^2_{E}$ and $\sigma ^2_{C}$ are portioned into an intercept and a slope parameter. This results in an estimate of $\sigma ^2_E$ and $\sigma ^2_C$ that is different for each twin pair *i*:

4 $$\sigma ^2_{Ei}= {\text {exp}}(\beta _0 + \beta _1 A_i)$$

5 $$\sigma ^2_{Ci}= {\text {exp}}(\gamma _0 + \gamma _1 A_i)$$

where $\beta _0$ and $\gamma _0$ denote the intercepts (i.e., unique-environmental variance when $A = 0$ and common-environmental variance when $C = 0$) and $\beta _1$ and $\gamma _1$ denote linear interaction effects (measuring A × E and A × C respectively), allowing that the environmental variance components are larger at either higher or lower levels of the genotype. The direction of both interaction effects depends on the sign of the interaction parameters, $\beta _1$ and $\gamma _1.$The exponential function was used to avoid negative variances (c.f. SanChristobal-Gaudy et al. 1998; Bauer and Hussong 2009; Hessen and Dolan 2009; van der Sluis et al. 2006; Molenaar et al. 2012).

To take into account properties of the measurement scale, simultaneously with the variance decomposition, the latent phenotype $\theta _{ij}$ appeared in the GPCM (for three response categories) for observed item data on item *k* of twin *j* from family *i*, $Y_{ijk}$. This was based on the results of the first part of the study, which suggested that this model was the best fitting IRT model for our data. Let $p_{ijkl}$ be the conditional probabilities of a particular response $l \in \left\{{\text{``no''}},{\text {``?''}},{\text{``yes''}}\right\}$ to an item *k* by twin member *j* from twin pair *i*, given the latent variables $\theta _{ij}$ and item parameters:

6 $$p(y_{ijk}={\text {``no''}}| \theta _{ij}, \alpha _k, \beta _{kl})=  \left[ 1+ e^{\alpha _k( \theta _{ij} - \beta _{k1} )} e^{\alpha _k( \theta _{ij} - \beta _{k2} )} \right]^{-1}$$

7 $$p(y_{ijk}={\text {``?''}}| \theta _{ij}, \alpha _k, \beta _{kl} )=  p(y_{ijk}={\text {``no''}}|.) \times e^{\alpha _k( \theta _{ij} - \beta_{k1})}$$

8 $$p(y_{ijk}={\text {``yes''}}| \theta _{ij}, \alpha _k, \beta _{kl} )= p(y_{ijk}={\text {``no''}}|.) \times p(y_{ijk}\quad ={\text{``?''}}|.) \times e^{\alpha _k( \theta _{ij} - \beta _{k2})}$$

where $\alpha _k$ is the discrimination parameter for item *k* (factorloading) and $\beta _{kl}$ is the *l*th threshold parameter for item *k*, representing the item “difficulties” that an individual has to “step through” in order to reach the next response category. The conditional probabilities can be intuitively interpreted as though an individual twin “passes through” each of the preceding answer categories before finally stopping at one response category (Li and Baser 2012). In order to identify the model, we assumed the first threshold to be zero for all items *k*, $\beta _{k1} = 0$, the phenotypic mean $\mu$ to be zero and set $\alpha _3$ to one. We estimated the thresholds for response categories “?” and “yes'', $\beta _{k2}$ and $\beta _{k3}$. Observed item data, $Y_{ijk}$ was assumed to have a multinomial distribution:

9 $$Y_{ijk} \sim Multinomial(p(y_{ijk} = y | \theta _{ij}, \alpha _k, \beta _{kl}))$$

The model is similar for DZ twins, but the genetic covariance in MZ twins is twice as large as in DZ twins. To model these different genetic correlations among MZ and DZ twins, first a normally distributed additive genetic effect *A*1 $VAR(A_1) = VAR(A_2) = \frac{1}{2} \sigma ^2_A$ was modelled and then, for each individual twin *j* from DZ pair *i*, a normally distributed additive genetic effect *A*2 was assumed. Furthermore, in order to model common-environmental influences *C*, we used a standard normal distribution. We then have for DZ twins:

10 $$A1_i\sim N\left(0, \frac{1}{2} \sigma ^2_A\right)$$

11 $$A2_{ij}\sim N\left(A1_i, \frac{1}{2} \sigma ^2_A\right)$$

12 $$C_i\sim  N(0,1)$$

In order to model A × C, the common-environmental effect *C* was scaled by multiplying it with the standard deviation $\sigma _{Cij}$, where $\sigma ^2_{Cij} = {\text {exp}}(\gamma _0 + \gamma _1 A2_{ij})$, yielding a common environmental effect *C*2 that was unique for every individual twin *j* from DZ pair *i*:

13 $$C2_{ij} = C_i \sqrt{{\text {exp}}(\gamma _0 + \gamma _1 A2_{ij}) }$$

To model A × E, the residual term was different for every individual twin:

14 $$\sigma ^2_{Eij} = {\text {exp}}(\beta _0 + \beta _1 A2_{ij})$$

A variance decomposition on the latent conservatism variable $\theta _{ij}$ for individual twin *j* from DZ pair *i* then yields:

15 $$\theta _{ij} \sim N( A2_{ij} + C2_{ij}, \sigma ^2_{Eij})$$

As for MZ twins, simultaneous to the variance decomposition, the latent phenotype, $\theta _{ij}$, appeared in the GPCM IRT model for three response categories (see Equations 6-8) and observed item data was assumed to have a multinomial distribution (see Equation 9).

*Prior distributions*

As prior distribution for the additive genetic variance, we chose an inverse gamma distribution ($\sigma ^2_A \sim InvG(1, 1)$). We chose independent normal distributions for both intercepts (${\text {exp}}(\beta _0)$, ${\text {exp}}(\gamma _0) \sim N(-1,2)$) as well as for both slope parameters ($\gamma _1$, $\beta _1 \sim N(0,10)$). For the item thresholds, we used a normal distribution $(\beta _k \sim N(0,10))$ and a lognormal distribution for the item discrimination parameters $({\text {log}}(\alpha _k) = N(0, 10)$).

In order to find the biometric model that fits the data well and, at the same time, is parsimonious, we estimated different biometric models. These included a biometric model without any interaction effects (simple ACE model), an ACE model with one (either A × E or A × C) interaction effect and a model with both interaction effects. The deviance information criterion (DIC, Spiegelhalter et al. (2002), a measure that estimates the amount of information that is lost when a given model is used to represent the data-generating process, was calculated to assess model fit of each model. The DIC takes account of both the complexity of a model and the goodness of fit. It can be seen as a Bayesian analog of Akaike’s Information Criterion (AIC). In the models without interaction effect(s), the same, independent, prior distributions were chosen for common-environmental and unique-environmental variance ($$\sigma ^2_C,$$$$\sigma ^2_E \sim InvG(1, 1)).$$

After a burn-in phase of 20,000 iterations for each separate chain, the characterisation of the posterior distribution for the model parameters was based on a total of 120,000 iterations from six different Markov chains. This was chosen on the basis of previous test runs with multiple chains and computing Gelman and Rubin’s convergence diagnostic (Gelman and Rubin 1992). The mean and standard deviation of the posterior point estimates was calculated for each parameter as was the 95 % highest posterior density (HPD, see e.g. Box and Tiao 1992) interval. The HPD can be interpreted as the Bayesian analog of a confidence interval (CI). When the HPD does not contain zero, the influence of a parameter can be regarded as significant.

*Sum score analysis*

In order to compare biometric results gained by this methodology with results gained by the sum score approach, the biometric model that was chosen as the best model for our data was also estimated using sum scores instead of item scores. In this analysis, sum scores were calculated from the twin data with answer categories coded as 0,1 and 2 respectively and re-scaled so that they had a mean of zero and variance of one in order to make results of both approaches comparable with respect to the prior distributions. Sum scores were then analyzed with the same JAGS script (see online supplementary material) but without the IRT part. After a burn-in period of 10,000 iterations, the characterisation of the posterior distribution for the sum score analysis was based on 15,000 iterations from 1 Markov chain, based on previous test runs with multiple chains and computing Gelman and Rubin’s convergence diagnostic (Gelman and Rubin 1992).

## Results

Based on the original 28 item scale with reverse coding (following Eaves et al. 1999), the reliability estimate was 0.73 (Guttman’s lambda 2; Cronbach’s alpha = 0.71). Item 28 (Busing) had a negative correlation (r = −0.18) with the total score, as did item 16 (Capitalism, r = −0.02).

### Homogeneity analysis results

**Fig. 1:**
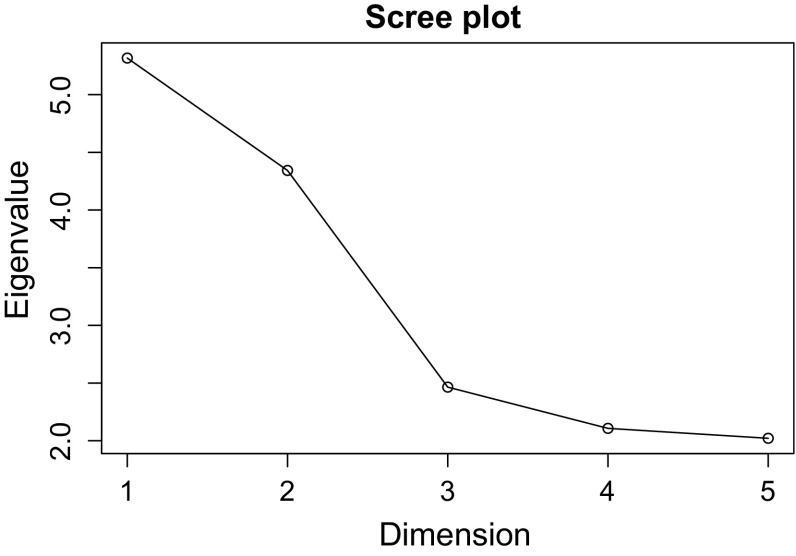
Homogeneity analysis: Scree plot that displays the eigenvalues associated with the first five dimensions

A homogeneity analysis was performed on the original item responses (no reverse coding). To evaluate the dimensionality of the scale, the eigenvalues associated with the first five dimensions were calculated, displayed in Fig. 1. It can be seen that, while the first two dimensions have a relatively large eigenvalue, the eigenvalues rapidly decrease when more dimensions are added to the scale.

**Fig. 2:**
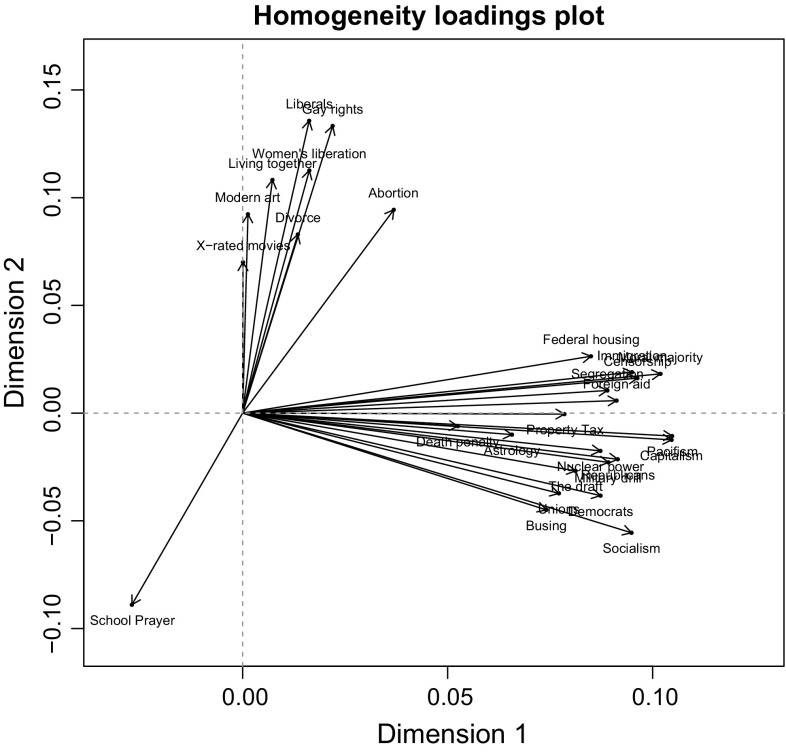
Plot of itemloadings based on a two-dimensional homals model

Based on these results, a model with two dimensions was chosen for further analysis. Item loadings on both dimensions can be seen in Fig. 2. We see that one item has a negative loading on dimension 2. Furthermore, a large number of items have strong and positive loadings on dimension 1, while a smaller subset of items have positive and strong loadings on dimension 2. Thus, some items mostly discriminate among individuals along the first dimension and some items discriminate among individuals along the second dimension.

**Fig. 3:**
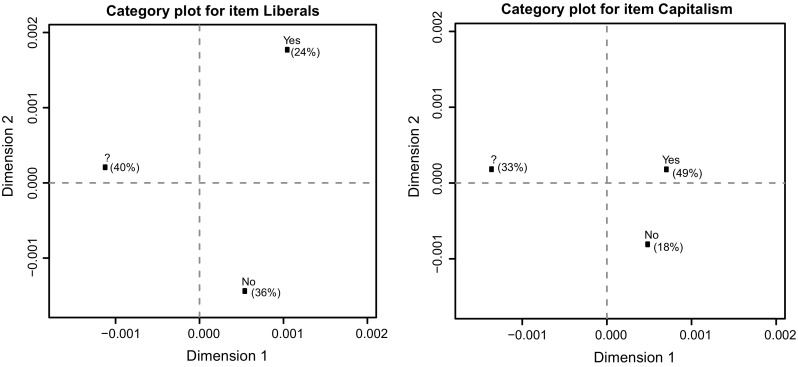
Category points plots for the catch-phrases “Liberals” (high loading on dimension 2) and “Capitalism” (high loading on dimension 1). Proportion of “yes”, “?” and “no” answers were added to the plots

In order to interpret the two dimensions, we plotted category points for the “yes”, “no” and “?” answer categories, which can be found in Fig. 3. To save space, the category points plots of only two items are displayed, but category points plots for all items can be found in the online supplementary material. A category point can be seen as the centre of gravity of all individuals who gave that particular response to a catch-phrase. When the category points are far apart on the x-axis (y-axis), this means that this item discriminates well between individuals on the first dimension (second dimension).

Investigating the category points on the second dimension of the catch-phrase “Liberals”, an item with a higher loading on the second dimension, we can see that the “?” answer category falls roughly between the “yes” and “no” answer categories. This implies that the second dimension distinguishes between liberal and conservative persons, assuming that someone with a high latent trait value for liberalism would be more inclined to approve of liberals (i.e., by answering “yes”) while someone with a very low latent trait for liberalism would be more inclined to disapprove of liberals (i.e. by answering “no”). When we look at the first dimension, we can observe that the distance between the “yes” and “no” answer categories is very small, while their distance to the “?” category point is relatively large. This suggests that the first dimension mainly distinguishes between people who answered with “?” or with either “yes” or “no”. When we investigate the category points plot for the catch-phrase “Capitalism”, an item with a higher loading on the first dimension, we can see the same pattern, but also that, although the “?” falls roughly in between the “yes” and “no” answer categories on the second dimension, the distance between the “yes” and “no” answer categories is much smaller on this dimension (compared to the “Liberals” item) while the distance between the “?” category point and the “yes” and “no” category points is still large on the first dimension. This suggests that this item better distinguishes between people who answered with “?” and people who answered with “yes” or “no” than between conservative and liberal persons.

*Interpretation of the dimensions*

**Fig. 4:**
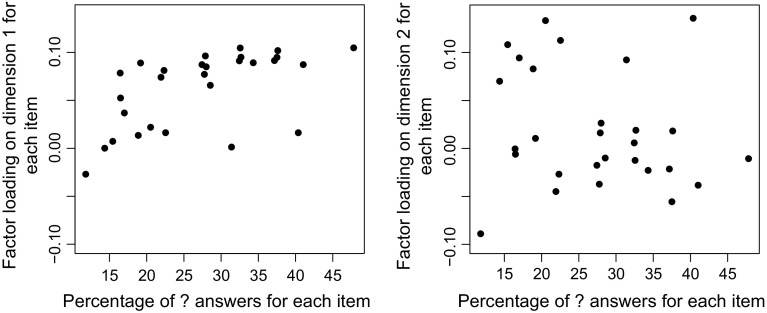
Item loadings on dimension 1 (*left*) and dimension 2 (*right*) for each item percentage of “?” answers for each item

The category points plots suggest that the second dimension can be interpreted as liberalism (positive direction)—conservatism (negative direction) dimension while the first dimension seems to distinguish mainly between respondents with a “?” answer and respondents with either a “yes” or “no” answer. To get more insight into the nature of the two dimensions, we looked at the relationship between the itemloadings and the proportion of “?” answers on each item, which can be seen in Fig. 4 for dimension 1 (left) and dimension 2 (right) separately. We can see that this relationship seems to be more or less random for the second dimension, but the proportion of “?” answers increases with higher itemloadings for dimension 1. A simple regression shows that, indeed, this relationship is positive and significant for the first dimension (T(26) = 3.75, *p*$<$ 0.01), but non-significant for the second dimension (T(26) = −0.936, *p* = 0.36).

The relationship between itemloadings and proportion of “?” answers on each item implies that the tendency of items to distinguish between individuals with a “?” answer and respondents who either answered “yes” or “no” is based in a high level of “?” responses, which might indicate that these items measure concepts on which many people simply do not have well-formed attitudes. The interpretation of this dimension however remains unclear, as we do not know participants’ true reason to give a “?” answer. Not having a well-formed attitude might indeed be the reason to give a “?” answer, but there are other possible reasons—for example, participants might not have understood what a particular catch-phrase means (e.g., item “Busing”).

*How can we handle this multidimensionality?*

A way to handle the multidimensionality of the Wilson−Patterson conservatism scale could be to use a weighted sum score, weighting items differently based on their itemloading on the second (liberalism-conservatism) dimension. However, although it is not possible to retrieve participants’ true motivation to give a “?” answer, category points plots as well as the relationship between factor loadings and the proportion of “?” answers suggest that items with a high loading on this dimension do not give much relevant information to distinguish between conservative and liberal persons. Therefore, we decided to use only items with a higher itemloading on dimension 2 (conservatism-liberalism) than on dimension 1 (ambiguous interpretation). For further psychometric analysis, we consequently selected the remaining 9 liberalism-conservatism items Gay rights, Women’s liberation, Living together, Modern art, Divorce, X-rated movies, School prayer (reverse-coded), Liberalism and Abortion. In all items, the “?” category point was in between the “yes” and “no” category points on the second dimension, a requirement for the application of the ordinal G(PCM) IRT model.

### Evaluation of the new scale

Item-total correlations showed positive signs and were in the range 0.50-0.68. The reliability was estimated at 0.78 (lambda 2).

A GPCM and a PCM were estimated using the 9 items. AIC and BIC favoured the GPCM over the PCM. Table 1 gives the estimated GPCM model parameters.

**Table 1:** GPCM parameter estimates and item fit statistics

	$\alpha$	$\beta _2$	$\beta _3$	$X^2$	*df*	*p*
X-rated movies	0.58	−1.52	−1.47	253.53	27	<0.01
Modern art	0.60	0.11	0.09	232.92	27	<0.01
Women’s liberation	1.00	0.22	0.80	204.93	27	<0.01
Abortion	1.01	−0.65	−0.16	112.60	27	<0.01
Gay rights	1.65	−0.90	−1.95	235.90	27	<0.01
Liberals	1.44	0.57	−0.67	357.63	27	<0.01
Living together	1.08	−0.91	−0.53	205.03	27	<0.01
Divorce	0.74	−0.44	0.23	147.16	27	<0.01
School prayer (rev. coded)	0.65	−1.86	−2.04	109.54	27	<0.01

Item fit statistics showed the largest χ^2^ value for the Liberals item. Observed number of responses for each response category as well as expected number of responses for each response category under the GPCM were plotted for ordered bins of total scores (see online supplementary material). Supplementary Fig. 1 shows that there is no systematic misfit for the Liberals item: the red lines (observed number of responses) largely overlap with the corresponding black lines (number of responses predicted by the fitted GPCM), as they do for all items. Supplementary Fig. 2 shows model fit based on twin data only.

### Biometric modelling

For the biometric modelling, only twin data were used. The DIC for all fitted biometric models can be found in Table 2. Based on these results, the model with an A × E effect as well as an A × C effect was chosen as the preferred model for our data.

**Table 2:** Model fit (DIC) for all fitted biometric models

Biometric model	DIC
No interaction effects (simple ACE model)	181853
ACE model with A × E	181782
ACE model with A × C	181849
ACE model with A × E and A × C	181776

*DIC* deviance information criterion

The results based on the model with an A × E and an A × C interaction effect are displayed in Table 3. The results suggest substantial A and C components, as well as a negative A × E interaction effect such that individuals having low genotypic values for liberalism show *more* residual variance than individuals with high genotypic values for liberalism. The HPD interval shows that this effect is significant. Furthermore, a significant and positive A × C interaction effect was found such that individuals having low genotypic values for liberalism show *less* common environmental variance than individuals with a high genotypic value for liberalism.

**Table 3:** Posterior mean (standard deviation) and HPD of variance components for the ACE model with A × E and A × C interaction effects

	$\sigma ^2_A$	${\text {exp}}(\gamma _0)$	${\text {exp}}(\beta _0)$	$\beta _1$	$\gamma _1$
Mean (SD)	0.43 (0.04)	0.29 (0.03)	0.07 (0.01)	−2.81 (0.21)	0.54 (0.14)
HPD	[0.33; 0.51]	[0.22; 0.35]	[0.05; 0.10]	[−3.22; −2.39]	[0.31; 0.84]

**Fig. 5:**
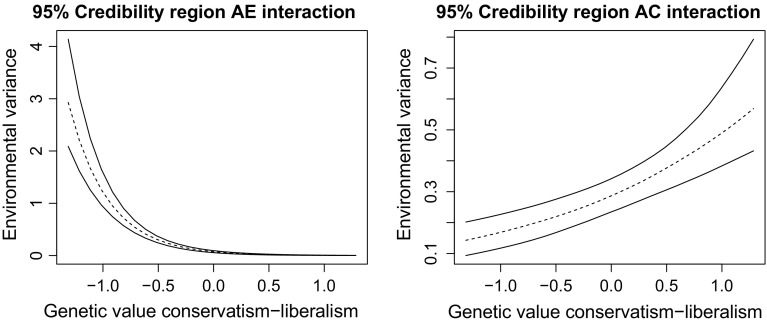
95 % credibility region for A × E interaction (*left*) and A × C interaction (*right*), separately for each genetic value. Based on the results of the ACE model with A × E and A × C

The 95 % credibility region for both interaction effects is displayed in Fig. 5 for the entire range of estimated genotypic values. Defined as $\frac{\sigma ^2_A}{\sigma ^2_P}$ where $\sigma ^2_P = \sigma ^2_A + {\text {exp}}(\gamma _0) + {\text {exp}}(\beta _0)$, the ACE model with A × E and A × C interaction effects leads to a narrow-sense heritability estimate $h^2$ of 0.43 (HPD: [0.34; 0.51]) with a standard deviation of 0.04.

*Analysis of sum scores*

**Fig. 6:**
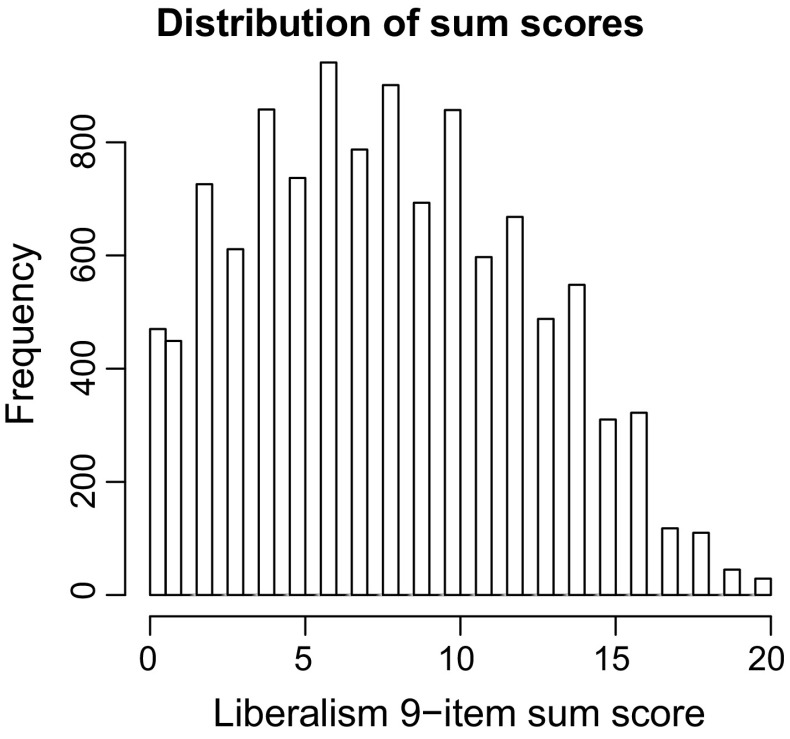
Distribution of sum scores of all MZ and DZ twins on the 9-item liberalism scale, with School prayer reverse-coded. Item categories were coded as 0, 1 and 2 respectively

Figure 6 shows the distribution of sum scores in twins. The results of the sum score approach can be found in Table 4. The sum score approach leads to a narrow-sense heritability $h^2$ of 0.42 (HPD: [0.38; 0.46]) with a standard deviation of 0.02. Note that, conversely to the results gained by the new methodology, the sum score approach found a non-significant *positive* A × E interaction effect and a significant *negative* A × C interaction effect.

**Table 4:** Sum score analysis: posterior mean (standard deviation) and HPD of variance components for the ACE model with A × E and A × C interaction effects

	$\sigma ^2_A$	${\text {exp}}(\gamma _0)$	${\text {exp}}(\beta _0)$	$\beta _1$	$\gamma _1$
Mean (SD)	0.64 (0.03)	0.35 (0.03)	0.53 (0.02)	0.10 (0.05)	−2.21 (0.09)
HPD	[0.58; 0.70]	[0.29; 0.41]	[0.49; 0.56]	[0.00; 0.21]	[−2.38; −2.05]

## Discussion

In this paper, we evaluated the Wilson−Patterson conservatism scale psychometrically before using a shorter version of the scale for a biometric analysis including genotype–environment interaction.

A psychometric evaluation of the 28-item conservatism scale Eaves et al. (1999) showed that this scale actually measures two different aspects in people: while one set of items distinguished between people’s agreement with either conservative or liberal catch-phrases, another set of items distinguished mainly between people who answered “?” or either “yes” or “no”. Earlier political research (e.g. Campbell et al. 1960; Converse 1964) has shown that most Americans are uninformed about politics and are not consistent in their agree- or disagreement with political statements. Arguably, it is likely that the dimension that differentiated mostly between respondents who answered “?” or either “yes” or “no”, mainly distinguished between individuals with and without an opinion. Considering the item content of the two sets of items, this seems plausible. While this dimension mainly consists of politically-loaded items (e.g., “Property Tax”, “Pacifism”) and seems to measure economic liberalism, the conservatism-liberalism dimension is composed of more approachable items (e.g., “Gay Rights”, “Abortion”), likely measuring social liberalism. Exceptions are the item “Liberals” on the conservatism-liberalism dimension and the items “Astrology” and “Death Penalty” on the dimension that distinguished between individuals who answered “?” or either “yes” or “no”.

To handle this multidimensionality, we decided to use a shorter version of the scale, consisting of only 9 items with a high loading on the liberalism-conservatism dimension. Ignoring the multidimensionality of the Wilson−Patterson conservatism scale can threaten validity of future or existing studies that use this scale. Therefore, we advise researchers to use the 9-item subscale as presented here rather than the full 28 items scale. Furthermore, results from the homogeneity analysis suggest that the item schoolprayer should be reverse-coded. An IRT analysis of this 9-item subscale showed good fit with a Generalized Partial Credit model and the reliability of the new scale was sufficient. The 9-item subscale is, however, constrained in the sense that the content of the remaining items reflects a measure of social liberalism rather than economic liberalism or security attitudes.

Comparing model fit and parsimony of different biometric models, an ACE model with both A × E and A × C was chosen as the best model for the data of this study. A biometric analysis that included an IRT model to correct for bias due to category response frequencies suggested a *negative* A × E and a *positive* A × C: a higher genetic propensity towards liberalism was associated with *less* unique-environmental and *more* common-environmental variance. The finding of a negative A × E effect means that the non-shared environment plays a more important role in explaining differences in individuals with a genetic tendency towards favouring conservative ideas than explaining differences in individuals genetically predisposed towards favouring liberal ideas. Conversely, the finding of a positive A × C effect means that the shared environment seems to be more important in explaining differences in individuals predisposed towards liberalism than in explaining differences in individuals predisposed towards conservatism. Arguably, genetic effects important for the expression of conservatism do not work in isolation, but instead influence the extent to which individuals are sensitive to environmental influences, favouring an interactionist framework for the study of conservatism as a personality trait.

These findings suggest that there are unique environmental factors that affect attitudes in the conservative genotype but much less affect attitudes in the liberal genotype. Likewise, the familial environment seems to be more important in forming political attitudes in families with a genetic tendency for liberalism than in families with a genetic tendency for conservatism. These results are surprising. Conservative people are generally seen as people who do not like change; they generally favour the safety of the known over the unknown (Wilson^1973^). Research by Carney et al. (2008) showed that two Big Five personality traits differentiate between liberal and conservative individuals: Openness to New Experiences and Conscientiousness. In general, conservative participants score higher on conscientiousness (e.g. being more conventional, orderly and better organized) whereas liberals score higher on openness to new experiences (e.g. being more curious, novelty-seeking and creative). The differences in personalities were even reflected in personal possessions and the characteristics of living and working spaces: Liberal participants collected more CDs, books, movie tickets, and travel paraphernalia, whereas conservative participants showed more sports decor, U.S. flags, cleaning supplies, calendars, and uncomfortable furniture. Based on these trait differences, one could expect that family environmental influences would be more important for individuals with a genetic tendency for conservatism than for individuals with a genetic tendency for liberalism. Likewise, it could be expected that unique-environmental influences would be more important for individuals genetically predisposed towards liberalism—with a tendency for novelty-seeking behaviour. Liberalism has been shown to be associated with higher IQ scores (Kanazawa 2010), which predicts that conservative people generally end up in different environmental circumstances than liberal-minded people and perhaps different amounts of variation of those environmental factors that act on political views and personality. The finding of a negative A × E suggests that conservatives might come into contact with people and ideas outside of their shared environment that might be more reflective of their genetic preference. Eaves et al. (1997) indeed showed that genetic expression of conservatism-liberalism only occurs after individuals have left their parental home. Individuals with a genetic tendency for conservatism then seem to be likely to be influenced by unique-environmental influences that might affect their thinking about political issues, while, surprisingly, individuals with a genetic tendency towards liberalism, are still influenced by their family environment. Future research on genotype–environment interaction in conservatism should focus on the exact nature of both, common and unique, influences by including specific, environmental moderators, measured at the family and individual level. This can be done, for example, by using the genotype-environment parametrization introduced by Purcell (2002) by regressing moderators directly on the genotypic value.

In order to compare results gained by the new methodology with the sum score approach, the same biometric model was estimated using sum scores instead of item scores. As the sum score approach does not take into account measurement unreliability, estimated average environmental variance was much higher. Furthermore, the sum score approach suggested a positive A × E and a negative A × C interaction effect, meaning that people with a genetic tendency towards liberalism show more residual variance and less common-environmental variance than people with a genetic tendency towards conservatism. However, since the distribution of sum scores was skewed, this may be an artefact of item characteristics (see e.g. Schwabe and van den Berg 2014; Molenaar and Dolan 2014).

To our knowledge, this is the first study that used the Wilson−Patterson scale to investigate genotype–environment interaction in case of unmeasured environmental variables. Regarding the testing of genotype–environment interaction in future research, we advise researchers to use the same IRT model (i.e., the GPCM) to make results concerning any interaction effects comparable. Results regarding genotype–environment interaction replicate only when the same underlying scale is used, as every transformation leads to a different result (see also Schwabe and van den Berg 2014; Molenaar and Dolan 2014).

In this research, the psychometric evaluation of the scale was done on all available data, of parents and offspring, enhancing statistical power. For the biometric modelling, however, only twin data was used. Unfortunately it was not possible to use a parent-offspring model for this paper, since methodology for the inclusion of a genotype–environment interaction effect in parent-offspring design with significant spouse correlation is still lacking. In future research, the method that was used in this paper will be extended to the parent-offspring design.

## Electronic supplementary material

Below is the link to the electronic supplementary material.

Supplementary material 1 (PDF 2305 kb)

## References

[CR1] Bauer D, Hussong A (2009). Psychometric approaches for developing com-mensurate measures across independent studies: traditional and new models. Psychol Methods.

[CR2] Boardman J (2011). Gene-environment interplay for the study of political be-haviors. Man is by nature a political animal.

[CR3] Bouchard T, Segal N, Tellegen A, McGue M, Keyes M, Krueger R (2003). Evidence for the construct validity and heritability of the wilson-patterson conservatism scale: a reared-apart twins study of social attitudes. Personal Individ Differ.

[CR4] Box GEP, Tiao GC (1992). Bayesian inference in statistical analysis.

[CR5] Cameron N (1993). Methodologies for estimation of genotype with environment interaction. Livest Prod Sci.

[CR6] Campbell A, Converse P, Miller W, Stokes D (1960). The american voter.

[CR7] Carney D, Jost J, Gosling S, Potter J (2008). The secret lives of liberals and conservatives: personality profiles, interaction styles, and the things they leave behind. Political Psychol.

[CR8] Caspi A, McClay J, Moffitt TE, Mill J, Martin J, Craig IW, Poulton R (2002). Role of genotype in the cycle of violence in maltreated children. Science.

[CR9] Chalmers RP (2012) mirt: a multidimensional item response theory package for the R environment. J Stat Softw 48(6):1–29. http://www.jstatsoft.org/v48/i06/

[CR10] Converse P (1964). The nature of belief systems in mass publics (1964). Critical review. J Politics Soc.

[CR11] de Leeuw J, Mair P (2009) Gifi methods for optimal scaling in R: the package homals. J Stat Softw 31 (4):1–20. http://www.jstatsoft.org/v31/i04/

[CR12] Development core team R (2007) R: a language and environment for statistical computing (Computer software manual). Austria, Vienna. http://www.R-project.org

[CR13] Eaves L (2006). Genotype x environment interaction in psychopathology: fact or artifact?. Twin Res Hum Genet.

[CR14] Eaves L, Erkanli A (2003). Markov chain monte carlo approaches to analysis of genetic and environmental change and g x e interaction. Behav Genet.

[CR15] Eaves L, Heath A, Martin N, Maes H, Neale M, Kendler K, Corey L (1999). Comparing the biological and cultural inheritance of personailty and social attitudes in the virgina 30,000 study of twins and their relatives. Twin Res.

[CR16] Eaves L, Last K, Martin N, Jinks J (1977). A progressive appraoch to non-additivity and genotype-environmental covariance in the analysis of human differences. Br J Math Stat Psychol.

[CR17] Eaves L, Martin N, Heath A, Schieken R, Meyer J, Silberg J, Corey L (1997). Age changes in the causes of individual differences in conservatism. Behav Genet.

[CR18] Gelfand A, Smith A (1990). Sampling-based approaches to calculating marginal densities. J Am Stat Assoc.

[CR19] Gelman A, Carlin J, Stern H, Rubin D (2004). Bayesian data analysis.

[CR20] Gelman A, Rubin D (1992). Inference from iterative simulation using multiple sequences. Stat Sci.

[CR21] Geman S, Geman D (1984). Stochastic relaxation, gibbs distributions and the bayesian restoration of images. IEEE Trans Pattern Anal Mach Intell.

[CR22] Gifi A (1990). Nonlinear multivariate analysis.

[CR23] Hatemi P, Funk C, Medland S, Maes H, Silberg J, Eaves NML (2009). Genetic and environmental transmission of political attitudes over a life time. J Politics.

[CR24] Hatemi PK, Medland SE, Klemmensen R, Oskarsson S, Littvay L, Dawes CT, Verhulst B, McDermott R, Norgaard AS, Klofstad CA, Christensen K, Johannesson M, Magnusson PKE, Eaves LJ, Martin NG (2014). Genetic influences on political ideologies: twin analyses of 19 measures of political ideologies from five democracies and genome-wide findings from three populations. Behav Genet.

[CR25] Heiser W, Meulman J, Greenacre M, Blasius J, Kristof W (1994). Homogeneity analysis: exploring the distribution of variables and their nonlinear relationship. Corrspondence analysis in the social sciences: recent developments and applications.

[CR26] Henningham J (1996). A 12-item scale of social conservatism. Person Individ Differ.

[CR27] Hessen D, Dolan C (2009). Heteroscedastic one-factor models and marginal maximum likelihood estimation. Br J Math Stat Psychol.

[CR28] Hibbing JR, Smith K, Alford J (2014). Dierences in negativity bias underlie variations in political ideology. Behav Brain Sci.

[CR29] Hicks BM, DiRago AC, Iacono WG, McGue M (2009). Gene—environment interplay in internalizing discorders: consistent findings across six environmental risk factors. J Child Psychol Psychiatry.

[CR30] IBM (2013) Released 2013. ibm spss statistics for windows, version 22.0 (Computer software manual). Armonk (3-900051-07-0)

[CR31] Inbar Y, Pizarro D, Bloom P (2009). Conservatives are more easily disgusted than liberals. Cognit Emot.

[CR32] Jinks J, Fulker D (1970). Comparison of the biometrical genetical, mava, and classical approaches to the analysis of human behavior. Psychol Bull.

[CR33] Kanazawa S (2010). Why liberals and atheists are more intelligent. Soc Psychol Q.

[CR34] Li Y, Baser R (2012). Using r and winbugs to fit a generalized partial credit model for developing and evaluating patient-reported outcomes assessments. Stat Med.

[CR35] Lunn DJ, Thomas A, Best N, Spiegelhalter D (2000). A bayesian modeling framework: concepts, structure, and extensibility. Stat Comput.

[CR36] Martin N, Spector T, Snieder H, MacGregor A (2000). Gene-environment interaction and twin studies. Advances in twin and sib-pair analysis.

[CR37] Martin N, Eaves L, Heath A, Jardine R, Feingold L, Eysenck H (1986). Transmission of social attitudes. Proc Natl Acad Sci USA.

[CR38] Molenaar D, Dolan C (2014). Testing systematic genotype by environment interactions using item level data. Behav Genet.

[CR39] Molenaar D, van der Sluis S, Boomsma D, Dolan C (2012). Detecting specific genotype by environment interactions using marginal maximum likelihood estimation in the classical twin design. Behav Genet.

[CR40] Muraki E (1992). A generalized partial credit model: application of an em algorithm. Appl Psychol Meas.

[CR41] Pedhazur E, Schmelkin L (1991). Measurement, design, and analysis: an integrated approach.

[CR42] Plummer M (2003) Jags: a program for analysis of bayesian graphical models using gibbs sampling

[CR43] Plummer M (2013). rjags: Bayesian graphical models using mcmc (Computer software manual). http://CRAN.R-project.org/package=rjags (R package version 3-10)

[CR44] Purcell S (2002). Variance components models for gene-environment interaction in twin analysis. Twin Res Hum Genet.

[CR45] SanChristobal-Gaudy M, Elsen J, Bodin L, Chevalet C (1998). Prediciton of the response to a selection for canalisation of a continous trait in animal breeding. Genet Sel Evol.

[CR46] Schreiber D, Fonzo G, Simmons A, Dawes C, Flagan T, Fowler J, Paulus M (2013). Red brain, blue brain: evaluative processes differ in democrats and republicans. PLoS ONE.

[CR47] Schwabe I, van den Berg S (2014). Assessing genotype by environment interaction in case of heterogeneous measurement error. Behav Genet.

[CR49] Sorensen D (2010) The genetics of environmental variation. In: Proceedings of 9th world congress on genetics applied to livestock. Leipzig

[CR50] Spiegelhalter D, Best N, Carlin B, van der Linde A (2002). Bayesian measures of model complexity and fit. J R Stat Soc.

[CR51] Turkheimer E, Haley A, Waldron M, D’Onofrio B, Gottesman II (2003). Socioeconomic status modifies heritability of iq in young children. Psychol Sci.

[CR52] van den Berg S, Beem L, Boomsma D (2006). Fitting genetic models using winbugs. Twin Res Hum Genet.

[CR53] van den Berg S, Glas C, Boomsma D (2007). Variance decomposition using an irt measurement model. Behav Genet.

[CR54] van den Berg S, Service S (2012). Power of irt in gwas: successful qtl mapping of sum score phenotypes depends on interplay between risk allele frequency, variance explained by the risk allele, and test characteristics. Genet Epidemiol.

[CR55] van der Kloot W (1997). Meerdimensionele schaaltechnieken voor gelijkenis- en keuzedata.

[CR56] van der Sluis S, Dolan C, Neale M, Boomsma D, Posthuma D (2006). Detecting genotype-environment interaction in monozygotic twin data: comparing the jinks and fullker test and a new test based on marginal maximum likelihood estimation. Twin Res Hum Genet.

[CR57] van der Sluis S, Verhage M, Posthuma D, Dolan C (2010). Phenotypic complexity, measurement bias, and poor phenotypic resolution contribute to the missing heritability problem in genetic association studies. Plos One.

[CR58] Wilson G (1973). The psychology of conservatism.

[CR59] Wilson G, Patterson J (1968). A new measure of conservatism. Br J Soc Clin Psychol.

