# Supplementary material for: Genes, Culture and Conservatism-A Psychometric-Genetic Approach
Source: Behav Genet. 2015 Nov 20;46:516–28. doi: 10.1007/s10519-015-9768-9 (PMC4886154; doi:10.1007/s10519-015-9768-9)
Supplement: Supplementary file 1 — Supplementary material 1 (PDF 2305 kb) [file 10519_2015_9768_MOESM1_ESM.pdf]

Genes, Culture and Conservatism - A Psychometric-Genetic Approach: Supplementary Material

Inga Schwabe Wilfried Jonker
Stéphanie M. van den Berg

This document contains supplementary material for the article "Genes, Culture and Conservatism - A Psychometric-Genetic Approach".

JAGS script

The JAGS (Plummer, 2003) script that was used for the biometric analysis described in the paper can be found at the end of this document. As similar syntax is used, the script can be used also in the free software package WinBUGS (Lunn, Thomas, Best, & Spiegelhalter, 2000) with minor adaptations. As an interface from R to JAGS, the rjags package was used (Plummer, 2013). The same script without the IRT part was used for the sum score analysis.

Homogeneity analysis: category point plots for all items

In order to interpret what the two dimensions represent, we plotted category points plots for the "yes", "no" and "?" answers. The category points plots for items with a high loading on the first dimension can be found in Figure 1 and Figure 2 and Figure 3 shows the category points plots for items with a high loading on the second dimension. Proportion of "yes"', '?" and "no" answers were added to all category points plots.

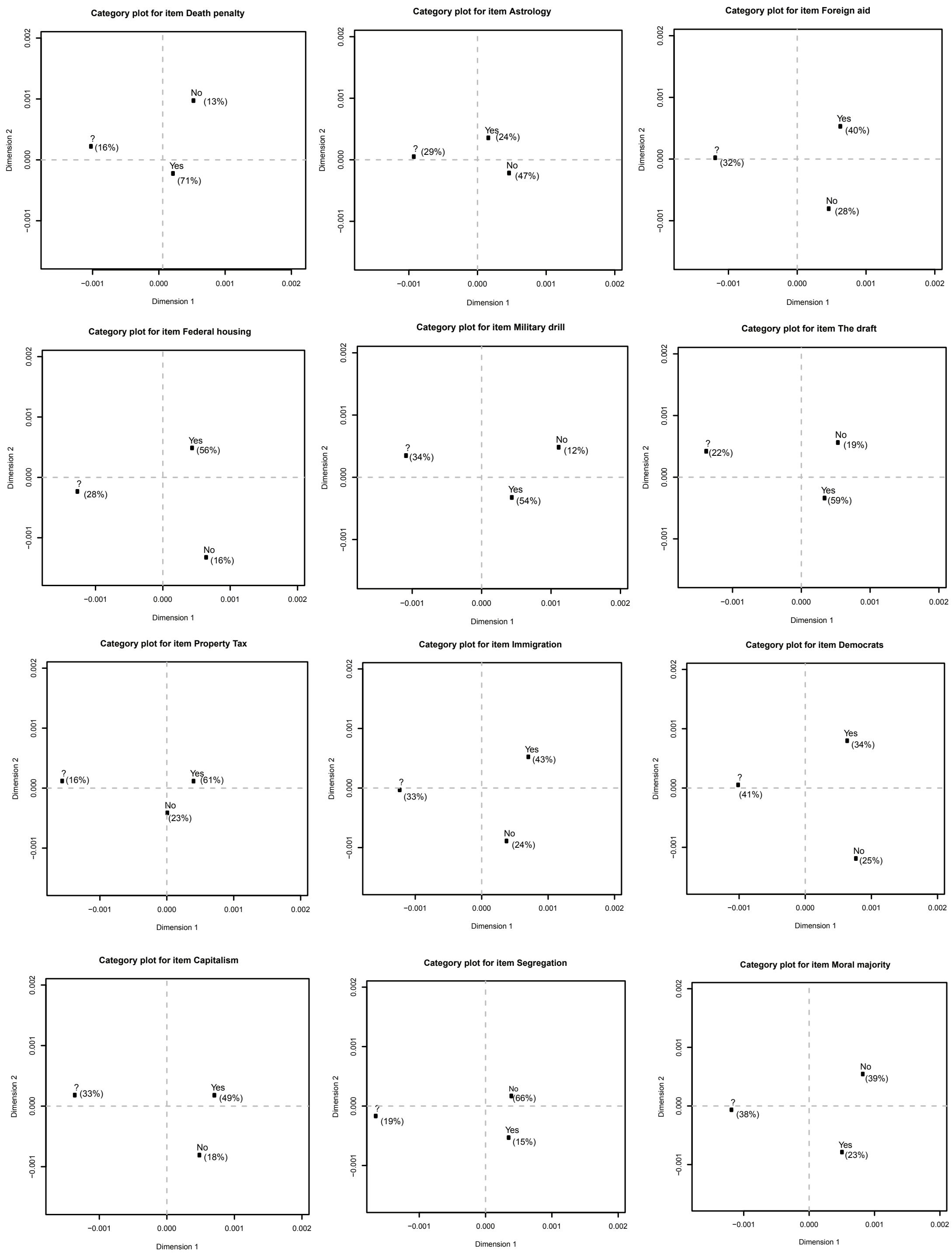

Figure 1: Category plots for items with high loadings on dimension 1 (part I).

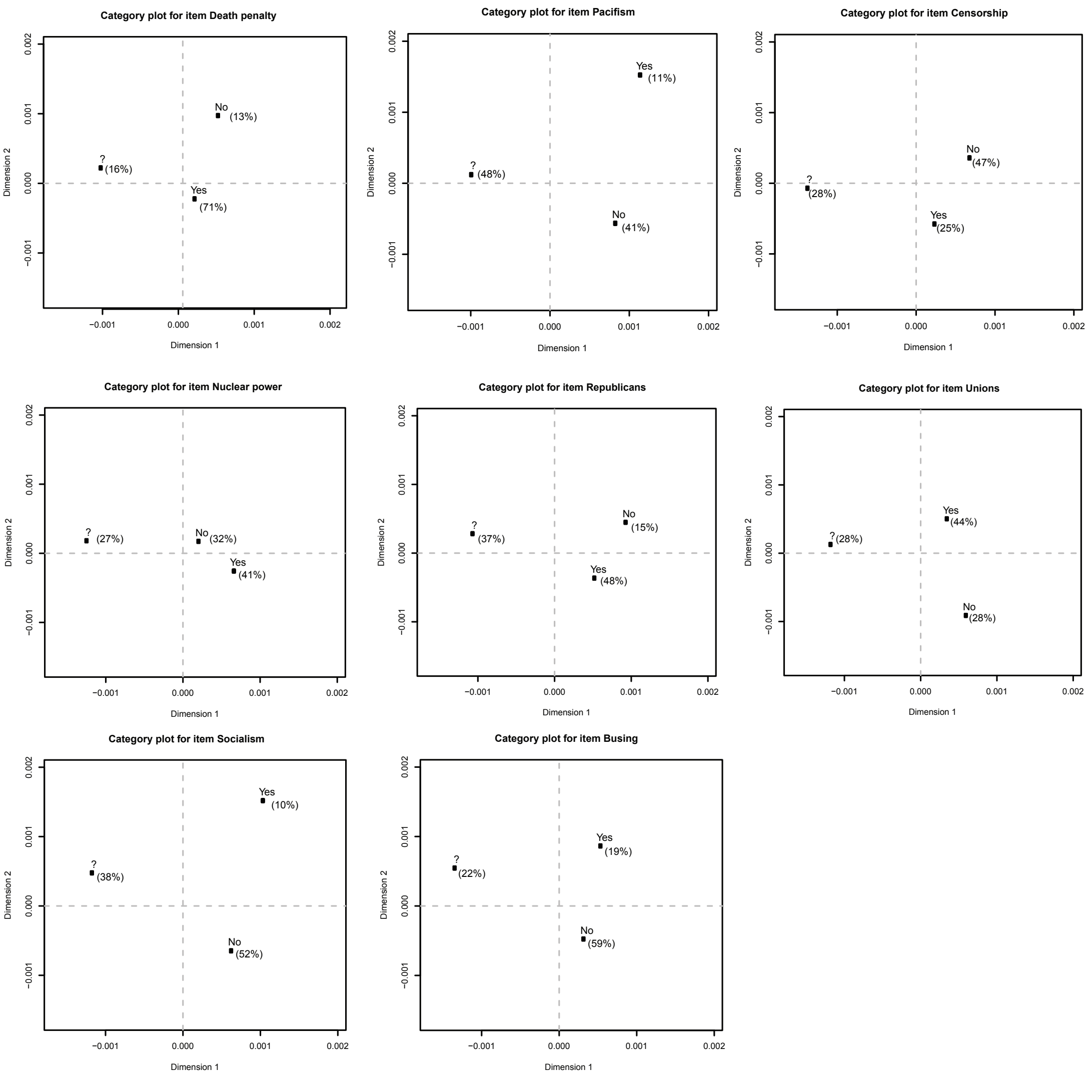

Figure 2: Category plots for items with high loadings on dimension 1 (part II)

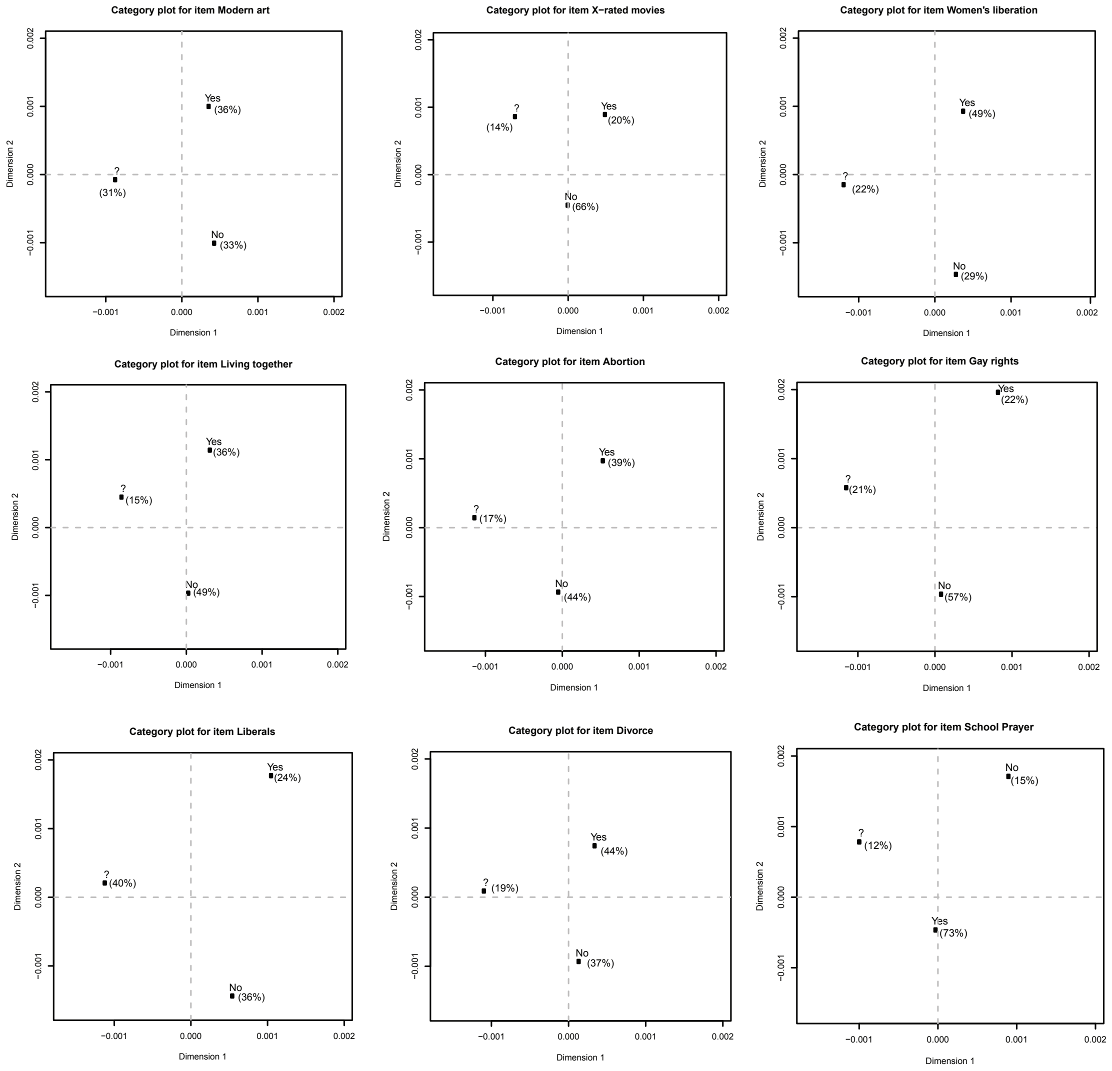

Figure 3: Category plots for items with high loadings on dimension 2.

Observed and expected number of responses

Observed number of responses for each response category as well as expected number of responses for each response category under the Generalized Partial Credit Model (GPCM) were plotted for ordered bins of total scores.

As stated in the manuscript, item fit statistics showed the largest chi-square value for the “Liberalism” item. Figure 4 however shows that there is no systematic misfit for this item: the red lines (observed number of responses) largely overlap with the corresponding black lines (number of responses predicted by the fitted GPCM), as they do for all items. Figure 5 shows model fit based on twin data only.

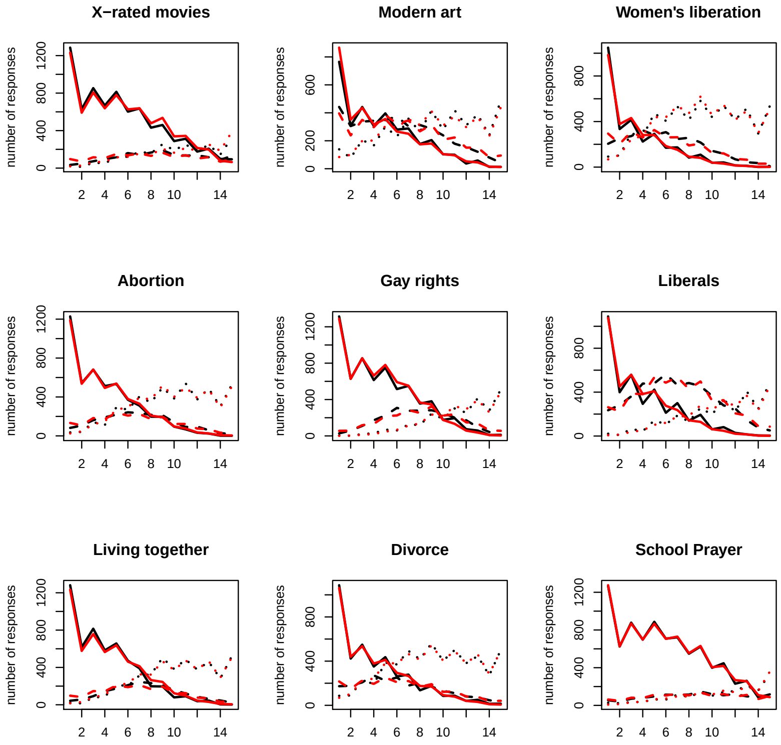

Figure 4: Observed (black) and expected (red) number of responses for each response category (“yes” = dotted line, “no” = solid line , “?” = dashed line), for ordered bins of total score (x-axis). Parent and twin data.

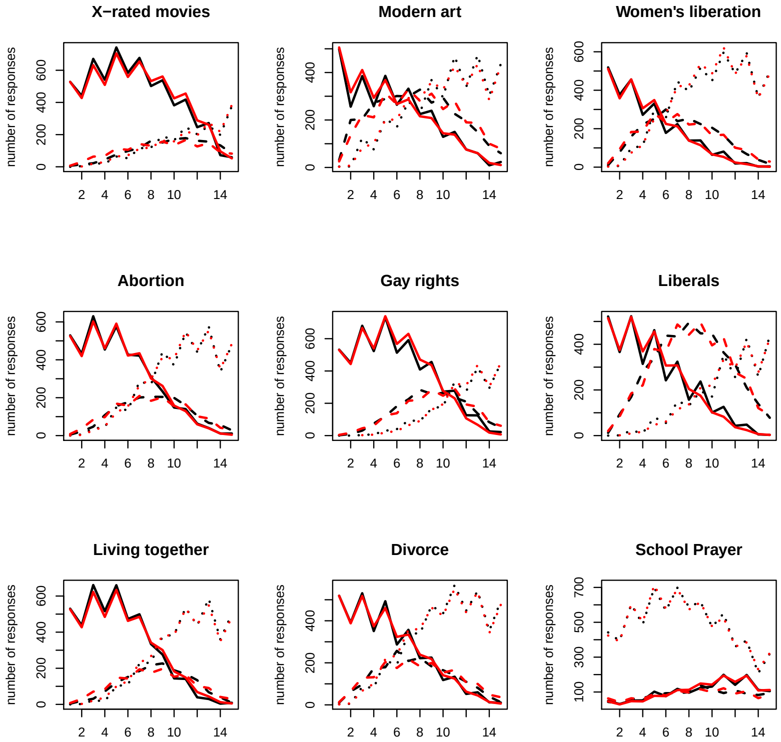

Figure 5: Observed (black) and expected (red) number of responses for each response category (“yes” = dotted line, “no” = solid line , “?” = dashed line), for ordered bins of total score (x-axis). Twin data only.

References

Lunn, D. J., Thomas, A., Best, N., & Spiegelhalter, D. (2000). A bayesian modeling framework: Concepts, structure, and extensibility. *Statistical Computing*, *10*, 325–337.

Plummer, M. (2003). *Jags: A program for analysis of bayesian graphical models using gibbs sampling.*

Plummer, M. (2013). rjags: Bayesian graphical models using mcmc [Computer software manual]. Retrieved from `http://CRAN.R-project.org/package=rjags` (R package version 3-10)

JAGS script

```
#Following script incorporates an ACE model with a generalized
#partial credit (GPCM) IRT model
#while modelling AxE and AxC genotype-environment interaction effects.

#y_dz = Item responses of DZ twins (matrix)
#y_mz = Item responses of MZ twins (matrix)
#n_mz = Number of MZ twin pairs,
#n_dz = Number of DZ twin pairs,
#n_items = Number of items administered

#Required structure of the y_dz/y_mz data matrix:
#y_dz[i,k] = kth datapoint from the ith DZ twin pair
#y_mz[i,k] = kth datapoint from the ith MZ twin pair

#This results in a matrix of n_mz (or, in case of y_dz, n_dz)
#rows and 2*n_items columns

#e.g. y_mz[1,22] is the response of MZ twin 1 from family 1 to item 22
#and y_mz[1,23] is the response of MZ twin 2 from family 1 to item 1
#if n_items = 22

#JAGS uses precision parameters for the variance parameters.
#Therefore, after running the script, these precision parameters
#should be inverted. For example:
#var_a <- 1/outputAnalysis$tau_a[,,1] with the rjags package

model{
#MZ twins
for (i in 1:n_mz){
        c_mz[i] ~ dnorm(0, tau_c_mz[i])
        a_mz[i] ~ dnorm(0, tau_a)

        tau_c_mz[i] <- 1/(exp(gamma0 + gamma1*a_mz[i]))
        tau_e_mz[i] <- 1/(exp(beta0 + beta1*a_mz[i]))

        #Phenotypic values:
        mz[i,1] ~ dnorm(a_mz[i] + c_mz[i], tau_e_mz[i])
        mz[i,2] ~ dnorm(a_mz[i] + c_mz[i], tau_e_mz[i])

        for (j in 1:n_items){
          for (k in 1:3){
                eta[i,j,k] <- alpha[j] * (mz[i,1]-beta[j,k])
                psum[i,j,k] <- sum(eta[i,j,1:k])
                exp_psum[i,j,k] <- exp(psum[i,j,k])
```

```
                prob[i,j,k] <- exp_psum[i,j,k]/sum(exp_psum [i,j,1:3])
          }
        }

          for (j in (n_items+1):(2*n_items)){
            for (k in 1:3){
                eta[i,j,k] <- alpha[j-n_items] * (mz[i,2]-beta[j-n_items,k])
                psum [i,j,k] <- sum(eta[i,j,1: k])
                exp_psum [i,j,k] <- exp( psum [i , j , k])
                prob [i,j,k] <- exp_psum[i,j,k]/sum(exp_psum [i,j,1:3])
          }
        }

          for (j in 1:(2*n_items)){
                y_mz[i,j] ~ dcat (prob[i,j,1:3]) # multinomial dist. data
        }

} #end MZ twins

#DZ twins
for (i in 1:n_dz){
          c_dz[i] ~ dnorm(0, 1)

          a1_dz[i] ~ dnorm(0, doubletau_a)
          a2_dz[i,1] ~ dnorm(a1_dz[i], doubletau_a)
          a2_dz[i,2] ~ dnorm(a1_dz[i], doubletau_a)

          tau_c_dz[i,1] <- exp(gamma0 + gamma1*a2_dz[i,1])
          tau_c_dz[i,2] <- exp(gamma0 + gamma1*a2_dz[i,2])

          c_dz_twin1[i] <- c_dz[i] * sqrt(tau_c_dz[i,1])
          c_dz_twin2[i] <- c_dz[i] * sqrt(tau_c_dz[i,2])

          tau_e_dz[i,1] <- 1/(exp(beta0 + beta1*a2_dz[i,1]))
          tau_e_dz[i,2] <- 1/(exp(beta0 + beta1*a2_dz[i,2]))

          dz[i,1] ~ dnorm(a2_dz[i,1] + c_dz_twin1[i], tau_e_dz[i,1])
          dz[i,2] ~ dnorm(a2_dz[i,2] + c_dz_twin2[i], tau_e_dz[i,2])

          for (j in 1:n_items){
            for (k in 1:3){
                etadz[i,j,k] <- alpha [j] * (dz[i,1]-beta [j,k])
                psumdz [i,j,k] <- sum(etadz[i,j,1: k])
                exp_psumdz[i,j,k] <- exp(psumdz [i,j,k])
                probdz [i,j,k] <- exp_psumdz [i,j,k]/sum(exp_psumdz[i,j,1:3])
          }
```

```
    }

        for (j in (n_items+1):(2*n_items)){
          for (k in 1:3){
                etadz[i,j,k] <- alpha[j-n_items] * (dz[i,2]-beta [j-n_items,k])
                psumdz [i,j,k] <- sum(etadz[i,j,1:k])
                exp_psumdz [i,j,k] <- exp(psumdz [i,j,k])
                probdz [i,j,k] <- exp_psumdz [i,j,k]/sum(exp_psumdz [i,j,1:3])
     }
    }

        for (j in 1:(2*n_items)){
                y_dz[i,j] ~ dcat (probdz[i,j,1:3]) # multinomial dist. data
    }
} #end DZ twins

#DZ twins genetic correlation 0.5:
doubletau_a <- 2*tau_a

#Set alpha of item 3 to 1 to identify the scale,
alpha[3] <- 1

#for the rest of the alpha parameters:
#lognormal prior with expectation of 0 (log(0) = 1) and variance of 10
alpha[1] ~ dlnorm(0, .1)
alpha[2] ~ dlnorm(0, .1)

for (j in 4:n_items){
    alpha[j] ~ dlnorm(0, .1)
}

#Beta IRT parameters: Use normal distribution with expectation of 0 and
#variance of 10:
for (j in 1:n_items){
    beta [j , 1] <- 0.0

    for (k in 2:3){
        beta [j , k] ~ dnorm (0, .1)
    }
}

#Priors for variance components,intercepts and interaction effects:
tau_a ~ dgamma(1,1)
beta0 ~ dnorm(-1,.5)
beta1 ~ dnorm(0,.1)
gamma0 ~ dnorm(-1,.5)
```

```
gamma1 ~ dnorm(0,.1)
}
```
